# Incidence Trends of Kaposi Sarcoma Among Young Non-Hispanic Black Men by US Regions, 2001-2018

**DOI:** 10.1093/jncics/pkac078

**Published:** 2022-11-10

**Authors:** Ryan Suk, Donna L White, Sheena Knights, Ank Nijhawan, Ashish A Deshmukh, Elizabeth Y Chiao

**Affiliations:** Department of Management, Policy and Community Health, The University of Texas Health Science Center School of Public Health, Houston, TX, USA; Center for Innovation, Quality, Effectiveness and Safety (IQuESt), Michael E. DeBakey VA Medical Center, Houston, TX, USA; Section of Health Services Research, Department of Medicine, Baylor College of Medicine, Houston, TX, USA; Section of Gastroenterology and Hepatology, Baylor College of Medicine, Houston, TX, USA; Division of Infectious Diseases and Geographic Medicine, Department of Internal Medicine, University of Texas Southwestern Medical Center, Dallas, TX, USA; Parkland Health and Hospital System, Dallas, TX, USA; Division of Infectious Diseases and Geographic Medicine, Department of Internal Medicine, University of Texas Southwestern Medical Center, Dallas, TX, USA; Parkland Health and Hospital System, Dallas, TX, USA; Hollings Cancer Center, Medical University of South Carolina, Charleston, SC, USA; Department of Public Health Sciences, Medical University of South Carolina, Charleston, SC, USA; Division of Cancer Prevention and Population Sciences, Department of Epidemiology, MD Anderson Cancer Center, Houston, TX, USA

## Abstract

Despite the overall national decline in Kaposi sarcoma (KS) incidence in the United States among persons living with HIV, previous studies suggest there might be specific subgroups of the US population that are associated with higher KS incidence rates than others. Using the 2001-2018 National Program of Cancer Registries and Surveillance, Epidemiology, and End Results Program database, we assessed KS incidence trends among young men aged 20-34 years by race and ethnicity and geographic region. Statistical significance is 2-sided. The KS incidence rate increased nationally by 1.5% per year in non-Hispanic Black men, whereas the rate decreased statistically significantly by 3.5% per year in non-Hispanic White men. A statistically significant 3.3% per year increase among young non-Hispanic Black men in the South and no change among those living in non-South regions were observed. Targeted HIV prevention and treatment in young non-Hispanic Black men in the South and further research addressing the increased KS incidence and burden in this vulnerable population are needed.

The advent and wide-scale dissemination of effective antiviral therapy has led to an overall national decline in Kaposi sarcoma (KS) incidence in the United States among persons living with HIV. Previous studies reported KS incidence trends across different racial and ethnic groups and regions. However, only partial information exists for fully understanding which subgroups are at an increasing risk of KS. A stable KS incidence trend among young Black people living with HIV was reported using 37 cancer registries. A study using the US Cancer Statistics showed an increasing trend among young Black men, whereas a rising trend in Black men of all ages living in the South was reported in a study using only 17 cancer registries (1-3). In this study, we sought to evaluate KS incidence and burden among young men stratified by race and ethnicity and geographic region to understand the possible existence of disparities.

Data are from population-based registries that participate in the Centers for Disease Control and Prevention’s National Program of Cancer Registries and/or the National Cancer Institute (NCI) Surveillance, Epidemiology, and End Results Program and meet high-quality data criteria. These registries cover approximately 98% of the US population during 2001-2018 (4). Incident KS was identified using the International Classification of Diseases for Oncology–3 code 9140. We calculated annual KS incidence rates and new cases (ie, burden) in young men aged 20-34 years by race and ethnicity abstracted from medical records (Hispanic, non-Hispanic Black, non-Hispanic White, and other [including American Indian and Alaska Native, Asian and Pacific Islander, and other races]), and geographic region (South, West, Northeast, and Midwest) using the Surveillance, Epidemiology, and End Results Program*Stat 8.3.9 (5). We assessed linear trends and derived average annual percentage changes (AAPC) in rates using the NCI Joinpoint 4.8.0.0 program (Supplementary Methods, available online) (6). We used NCI’s age-period-cohort analysis web tool to simultaneously evaluate age, calendar time period, and birth cohort effects on KS incidence, presenting incidence rate ratios (IRRs) by birth cohort (Supplementary Methods, available online) (7). For comparison, we selected the reference year corresponding to the 1979 birth cohort when presenting incidence rate ratios; this arbitrary choice of reference year does not affect result interpretation. Statistical significance was assessed at a statistical significance level of a *P* value less than .05, and all tests were 2-sided. This study was deemed exempt from review by the institutional review board of the University of Texas Health Science Center because it uses publicly available deidentified data.

During 2001-2018, a total of 3911 KS cases were diagnosed among men aged 20 to 34 years in the United States. For our analyses, we limited to 3838 KS cases with complete race and ethnicity information. Of those cases, 1800 (46.0%) cases were diagnosed in non-Hispanic Black men, 942 (24.1%) cases in non-Hispanic White men, 980 (25.1%) cases in Hispanic men, and 116 (3.0%) cases in other races. During this time, the KS incidence rate increased statistically significantly by 1.5% per year (95% confidence interval [CI] = 0.6% to 2.5%) in non-Hispanic Black men. In contrast, a rapid statistically significant decline (AAPC = −3.5%, 95% CI = −5.0% to −2.0%) was observed among non-Hispanic White men. Rates in Hispanic men did not change (AAPC = −1.3%, 95% CI = −11.2% to 9.7%) (Figure 1, A). The trend analysis for other races could not be conducted because of the small sample size. Geographic analyses demonstrated a statistically significant 3.3% per year (95% CI = 2.1% to 4.4%) increase among young non-Hispanic Black men in the South and no change among young non-Hispanic Black men living in non-South regions (AAPC = −0.9%, 95% CI = −2.4% to 0.7%) (Figure 1, B). There were no geographic differences among young non-Hispanic White men (Supplementary Figure 1, available online). Between 2001 and 2018, the contribution of the South to the national burden of KS among non-Hispanic Black men aged 20-34 years increased from 45% to 62%. The age-period-cohort analysis further demonstrated that non-Hispanic Black men born in recent years (the 1994 cohort) in the South had more than twofold higher KS risk (IRR = 2.2, 95% CI = 1.5 to 3.3) than the 1979 cohort (Figure 2, A). No statistically significant differences were observed between the 1994 birth cohort and the 1979 cohort in non-South regions (IRR = 1.4, 95% CI = 0.8 to 2.4) (Figure 2, B).

**Figure 1.**
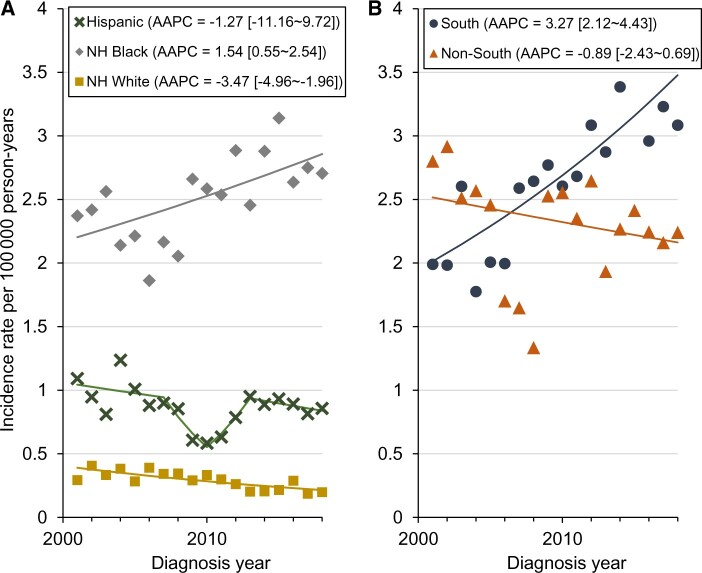
Incidence rate trends of Kaposi sarcoma in young men aged 20-34 years.**A**) National incidence rate trends. **B**) Regional incidence rate trends in non-Hispanic Black men. The trend analysis for other races (including American Indian and Alaska Native, Asian and Pacific Islander, and other races) could not be conducted because of the small sample size. **Square brackets** contain 95% confidence intervals. AAPC = average annual percentage change; NH = non-Hispanic.

**Figure 2.**
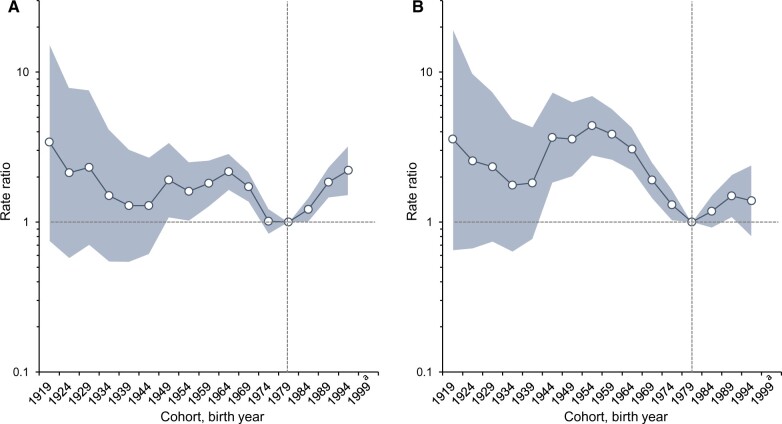
Age-period-cohort analysis of Kaposi sarcoma incidence in non-Hispanic Black men by region. **A**) Birth cohort effects in non-Hispanic Black men, South region. **B**) Birth cohort effects in non-Hispanic Black men, non-South region (West, Northeast, Midwest). ^a^Not reported because there were fewer than 16 cases in the time interval.

Our study over an 18-year contemporary period in the United States offers evidence supporting an ongoing rise in KS burden among the general subpopulation of young non-Hispanic Black men living in the South. Age-period-cohort analysis suggests that increasing KS risk is likely attributable to increasing exposure to KS risk factors, including HIV infection among the recent-born non-Hispanic Black men in the South. Notably, although the National Program of Cancer Registries and the NCI Surveillance, Epidemiology, and End Results Program registry data do not include HIV or AIDS status, prior research suggests the majority of KS cases in men aged 20-34 years were associated with HIV or AIDS (8). Therefore, it is likely that the observed increase in KS incidence in young non-Hispanic Black men in the South is primarily related to known parallel increases in HIV incidence in young, non-Hispanic Black men who have sex with men living in the South (9,10) and is also associated with a high prevalence of undiagnosed HIV infections in Black men (10). It has also been shown that the Southern states have high levels of poverty and HIV-related stigma (11), which are also likely to contribute to higher HIV-related KS incidence. Social and sexual network factors could also play a role as a driver of these racial and ethnic disparities of HIV incidence and likely KS incidence (12). KS incidence in the United States has shown overall decreasing rates among people living with HIV (2), however, these aggregated trends may mask trends in racial and geographic subgroups that have already been shown to have poorer HIV-related outcomes. Of note, recent studies utilizing the HIV and AIDS Match Cohort did not find any statistically significant increases in KS rates among any demographic or geographic group (2,13). Although those studies did not specifically evaluate the subpopulation of young non-Hispanic Black men living in southern states, their analyses used individuals reported in the state HIV registries as the denominator. Therefore, the impact of increasing HIV incidence in this population demonstrated by the current study would not be captured over time in HIV registry studies. Collectively, our findings underscore an ongoing disproportionately rising KS burden among young non-Hispanic Black men in the South likely attributable to a combination of the increasing incidence of HIV and AIDS, HIV-related stigma, lack of access to medical care, and sexual and/or social network dynamics. However, further research on the potential synergetic effects of HIV and KS-associated herpesvirus transmission is needed. The findings suggest a critical need for improving targeted HIV prevention and treatment, including focusing on the impact of stigma and access to medical care on KS incidence and burden in young non-Hispanic Black men in the South.

## Funding

R01 CA232888 by National Cancer Institute (Drs Deshmukh and Chiao); R01 CA260689 by National Cancer Institute (Dr Chiao).

## Notes

**Role of the funder:** The funder had no role in the design of the study; collection, analysis, and interpretation of the data; or the preparation of the manuscript or decision to submit it for publication.

**Disclosures:** AAD reported receiving consulting fees from Merck & Co, outside the submitted work. The other authors have no disclosures.

**Author contributions:** RS: Conceptualization; Data curation; Formal analysis; Investigation; Methodology; Project administration; Software; Validation; Visualization; Writing—original draft; Writing—review & editing. DLW: Conceptualization; Data curation; Investigation; Writing—original draft; Writing—review & editing. SK: Data curation; Investigation; Writing—original draft; Writing—review & editing. AN: Data curation; Investigation; Writing—original draft; Writing—review & editing. AAD: Conceptualization; Data curation; Investigation; Methodology; Project administration; Supervision; Validation; Visualization; Writing—original draft; Writing—review & editing. EYC: Conceptualization; Data curation; Funding acquisition; Investigation; Methodology; Project administration; Supervision; Validation; Visualization; Writing—original draft; Writing—review & editing.

**Disclaimer:** The content is solely the responsibility of the authors.

## Supplementary Material

pkac078_Supplementary_Data

## Data Availability

The US Cancer Statistics Databases (National Program of Cancer Registries [NPCR] and Surveillance Epidemiology and End Results [SEER]) are publicly available at https://www.cdc.gov/cancer/uscs/public-use/about.htm. Documentation for data use is also available at https://www.cdc.gov/cancer/uscs/public-use/us/index.htm. Data analysis information that supports the findings of this study is available from Dr Suk (ryan.suk@uth.tmc.edu) upon request.
