# Supplementary material for: Incidence Trends of Kaposi Sarcoma Among Young Non-Hispanic Black Men by US Regions, 2001-2018
Source: JNCI Cancer Spectr. 2022 Nov 10;6(6):pkac078. doi: 10.1093/jncics/pkac078 (PMC9703956; doi:10.1093/jncics/pkac078)
Supplement: pkac078_Supplementary_Data [file pkac078_supplementary_data.pdf]

SUPPLEMENTARY MATERIALS

Incidence trends of Kaposi Sarcoma among Young Non-Hispanic Black Men by US Regions, 2001-2018

Supplementary Methods

References

Supplementary Figure 1. Regional Kaposi sarcoma incidence rate trends in non-Hispanic White men aged 20-34

Supplementary Methods

Fitting the joinpoint regression

To identify the temporal trends and joinpoints (the calendar years where the trends change significantly), the NCI's Joinpoint program selected the best-fitting log-linear regression model, allowing for the minimum number of joinpoints necessary to fit the data.[1] The Joinpoint program performs multiple tests to select the number of joinpoints; the permutation test is used repeatedly for testing between two different joinpoint models, a simpler model with fewer joinpoints called the null model and a more complicated model called the alternative model. The annual percentage change (APC) characterizes a trend, a single regression line fitted over a fixed interval, whereas the average annual percentage change (AAPC) is a weighted average of the APCs from the joinpoint model with the weights equal to the length of the APC interval. For this study, we only report AAPCs because most of the groups of interest did not show a joinpoint (trend change) and therefore had a single APC that is the same as AAPC. To determine whether the trends differed significantly from 0, *t*-tests for APCs and z-tests for AAPCs were used (t-tests when the AAPC was within one APC segment). Statistical significance was assessed at an α level of $P<0.05$, and all hypotheses were 2-sided.

Age-period-cohort analysis

To simultaneously assess the effect of age, calendar period, and birth cohort on the KS incidence rate, we performed an age-period-cohort analysis using NCI's Age Period Cohort web tool.[2] For this analysis, we grouped 5-year age groups and periods with 5-year intervals to identify individuals who belong to approximately the same birth cohort (sixteen birth cohorts). Each cohort comprises ten birth years, with partially overlapping years with other adjacent birth cohorts.[3]

REFERENCES

1. Kim HJ, Fay MP, Feuer EJ*, et al.* Permutation tests for joinpoint regression with applications to cancer rates. Stat Med 2000;19(3):335-51.

2. Rosenberg PS, Check DP, Anderson WF. A web tool for age-period-cohort analysis of cancer incidence and mortality rates. Cancer Epidemiol Biomarkers Prev 2014;23(11):2296-302.

3. Rosenberg PS, Anderson WF. Age-period-cohort models in cancer surveillance research: ready for prime time? Cancer Epidemiol Biomarkers Prev 2011;20(7):1263-8.

Supplementary Figure 1. Regional Kaposi sarcoma incidence rate trends in non-Hispanic White men aged 20-34

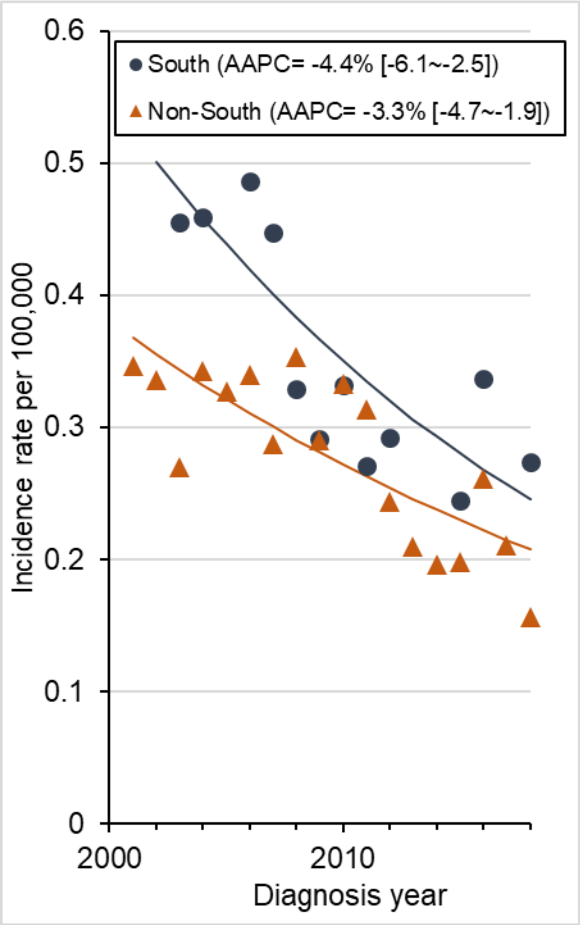

Abbreviation: AAPC, average annual percentage change

Square brackets contain 95% confidence intervals.
